# Genomic Prediction in Pea: Effect of Marker Density and Training Population Size and Composition on Prediction Accuracy

**DOI:** 10.3389/fpls.2015.00941

**Published:** 2015-11-17

**Authors:** Nadim Tayeh, Anthony Klein, Marie-Christine Le Paslier, Françoise Jacquin, Hervé Houtin, Céline Rond, Marianne Chabert-Martinello, Jean-Bernard Magnin-Robert, Pascal Marget, Grégoire Aubert, Judith Burstin

**Affiliations:** ^1^INRA, UMR1347 AgroécologieDijon, France; ^2^INRA, US1279 Etude du Polymorphisme des Génomes Végétaux, CEA-IG/Centre National de GénotypageEvry, France

**Keywords:** pea (*Pisum sativum* L.), GenoPea 13.2K SNP Array, genomic selection, marker density, training set, prediction accuracy

## Abstract

Pea is an important food and feed crop and a valuable component of low-input farming systems. Improving resistance to biotic and abiotic stresses is a major breeding target to enhance yield potential and regularity. Genomic selection (GS) has lately emerged as a promising technique to increase the accuracy and gain of marker-based selection. It uses genome-wide molecular marker data to predict the breeding values of candidate lines to selection. A collection of 339 genetic resource accessions (CRB339) was subjected to high-density genotyping using the GenoPea 13.2K SNP Array. Genomic prediction accuracy was evaluated for thousand seed weight (TSW), the number of seeds per plant (NSeed), and the date of flowering (BegFlo). Mean cross-environment prediction accuracies reached 0.83 for TSW, 0.68 for NSeed, and 0.65 for BegFlo. For each trait, the statistical method, the marker density, and/or the training population size and composition used for prediction were varied to investigate their effects on prediction accuracy: the effect was large for the size and composition of the training population but limited for the statistical method and marker density. Maximizing the relatedness between individuals in the training and test sets, through the CDmean-based method, significantly improved prediction accuracies. A cross-population cross-validation experiment was further conducted using the CRB339 collection as a training population set and nine recombinant inbred lines populations as test set. Prediction quality was high with mean *Q*^2^ of 0.44 for TSW and 0.59 for BegFlo. Results are discussed in the light of current efforts to develop GS strategies in pea.

## Introduction

New breeding challenges imposed by rapidly-expanding world population and global climate change urge crop breeders to develop and utilize more efficient selection approaches. Several breeding techniques exploiting high-density genotyping data have lately emerged in parallel with advances in sequencing technologies. Genomic selection (GS) is a revolutionary approach where a breeder's selection is made on the basis of genomic estimated breeding values (GEBVs) obtained from genome-wide DNA marker information (Meuwissen et al., 2001). GS also benefits from novel statistical methods that are proposed for data under large “p,” small “n” conditions and that so enable the simultaneous estimation of all marker effects (Jannink et al., 2010). Genotyping and phenotyping of a training population allow developing a prediction model that is then used to predict the performance of a genotyped, non-phenotyped test population and select superior individuals. Studies conducted on plant populations have provided evidence that GS can potentially increase the accuracy of marker-based selection and outperform phenotypic selection and conventional marker-assisted selection in terms of gain per unit time and cost (Heffner et al., 2011). Since the genetic gain is proportional to the prediction accuracy, prediction accuracy improvement is a major issue when applying GS. Prediction accuracy has been reported to depend on the genetic architecture of the trait (Jannink et al., 2010; Burstin et al., 2015), the number and the distribution of the genetic markers (Heffner et al., 2011; Poland et al., 2012; Heslot et al., 2013), and the size (Heffner et al., 2011; Jarquín et al., 2014) and composition of the training population (Rincent et al., 2012; Charmet et al., 2014).

Pea (*Pisum sativum* L.) is a cool-season legume crop grown essentially for its protein-rich seeds (Burstin et al., 2011) and as a valuable component of low-input farming systems. Pea selection has brought important innovations for several agronomic traits [see Tayeh et al. (2015), for review]. Improving resistance to biotic and abiotic stresses is now a major target for breeders, especially in the context of climate change. Genomic prediction has been evaluated in pea using 367 genetic resource accessions and 331 gene-based markers from an Illumina GoldenGate SNP assay (Burstin et al., 2015). New pea genomic resources including molecular markers, transcript sequences, and comprehensive genetic maps have been developed recently (Bohra et al., 2014; Tayeh et al., 2015). The GenoPea 13.2K SNP Array is the largest genotyping platform currently available with 13204 gene-based SNP markers (Tayeh et al., in press). It has been used to genotype 12 pea bi-parental mapping populations and subsequently construct a high-density consensus map comprising 12802 SNP markers and showing a cumulative length of 794.9 cM Haldane and a mean inter-marker distance of 0.24 cM (Tayeh et al., in press).

The present study aimed at re-evaluating genomic prediction in the pea genetic resource collection previously described in Burstin et al. (2015) using high-density genotyping information derived from the GenoPea 13.2K SNP Array. The effects on the genomic prediction accuracy of (i) the statistical prediction method, (ii) the density of the genetic markers, and (iii) the size of the training population and its composition were investigated. This work is expected to accelerate the implementation of GS in applied pea breeding programs.

## Materials and methods

A collection of 339 accessions (Supplementary Table 1), hereafter referred to as the CRB339 collection, was defined from the 372-accession pea genetic resource collection described in Burstin et al. (2015) and used in this study. Closely-related accessions were eliminated from the complete set so that the final genomic relationship matrix can be solved. Genotypes having no available phenotypic data, a marker missing rate ≥0.1, and/or an heterozygosity ≥0.05 were also discarded. The CRB339 collection was genotyped with the newly-developed custom Infinium BeadChip (Illumina, San Diego, CA) GenoPea 13.2K SNP Array as described in Tayeh et al. (in press). Data were analyzed using the Genotyping Module v1.9.4 of Illumina's GenomeStudio® software version 2011.1 (http://support.illumina.com/array/array_software/genomestudio.ilmn). When necessary, GenoPlots were manually edited so that three genotype clusters AA, AB, and BB could be obtained (Supplementary Figure 1). A qualitative score describing the clustering profile was given for each marker according to Supplementary Figure 1. Only data from markers showing high-quality GenoPlots (score 1; Supplementary Figure 1) and having been reported of high mapping quality on individual and consensus maps from bi-parental populations in Tayeh et al. (in press) were considered (*n* = 9824 markers). The CRB339 collection was used to test the effect of the statistical method, the marker density, and the training population size and composition on genomic prediction accuracy. The full list of markers used for genomic prediction is provided in Supplementary Table 3. Missing data were submitted to a k-nearest neighbor imputation (Troyanskaya et al., 2001) using a custom script. To visualize their distribution on the pea genome, markers were plotted on the consensus genetic map (Tayeh et al., in press) using the plotGenMap function from the synbreed package (Wimmer et al., 2012) of the R environment (R_Core_Team, 2015). Linkage disequilibrium (LD) *r*^2^ values were measured using the pairwise LD function from the same package and plotted against pairwise genetic distances between markers according to the consensus map (Tayeh et al., in press). The decay of *r*^2^ with distance was fitted using Hill and Weir's expectation of *r*^2^ between adjacent sites (Hill and Weir, 1988), similar as in Marroni et al. (2011). A set of 746 advanced recombinant inbred lines (RILs) derived by single-seed descent from nine different bi-parental crosses (Pop2-10; Supplementary Table 2) and also genotyped with the GenoPea 13.2K SNP Array (Supplementary Table 3; Tayeh et al., in press) was finally used as a test population in a cross-population cross-validation experiment to evaluate the prediction ability with the applied statistical methods and the effect of the training population on the accuracy of genomic predictions.

Phenotypic data for three traits, including the thousand-seed weight (TSW), the number of seeds per plant (NSeed), and the date of beginning of flowering (BegFlo; expressed as the sum of daily temperatures above 0°C between sowing and flowering of the first node), were collected from field trials at Dijon-Epoisses (47.23618N, 5.09796E) in 2003 and 2007 (see Burstin et al., 2015 for details) for the CRB339 population (Supplementary Table 1) and in 2011 for the RIL populations (Supplementary Table 2). The 2003 and 2007 field conditions were contrasted as regards to plant density and to climate (Burstin et al., 2015). As described in Burstin et al. (2015), the phenotypic scores from the two randomly-replicated block field trial of 2007 were analyzed by a two-way ANOVA with a genotype and a block effect to obtain mean phenotypic values for the considered accessions (proc GLM, SAS Institute). One-block design trials with replicated controls were performed in 2003 and 2011; the data for these 2 years were used to evaluate the cross-year and cross-population goodness of fit of the models based on the phenotyping of 2007, respectively.

Five different marker-based statistical prediction algorithms were applied for genomic prediction of the phenotypic traits: Kernel Partial Least Squares Regression (kPLSR), Least Absolute Shrinkage and Selection Operator (LASSO), Genomic Best Linear Unbiased Prediction (GBLUP), BayesA, and BayesB using the following R packages: pls (Mevik et al., 2012; kPLSR), glmnet (Friedman et al., 2010; LASSO), rrBLUP (Endelman, 2011; GBLUP), and synbreed (Wimmer et al., 2012; BayesA and BayesB). A probability (π) of 0.95 was used for BayesB. The number of latent variables k for kPLSR and the optimum value for the tuning parameter t in LASSO were determined through 10-fold cross-validations as the values minimizing the Mean Squared Error of Prediction (MSEP). Considering results from Burstin et al. (2015) indicating that structure correction did not improve the efficiency of phenotype prediction, no correction for the population structure effect was made in this study before applying the statistical methods.

The training population size and the number of markers were varied to investigate their effects on prediction accuracy via cross-validation following 200 repetitions. Within each repetition, the training population size was varied as follows: 240, 210, 180, 150, 120, 90, 60, 30, and 15 accessions. The same test set was used with all training populations. The training sets were randomly selected and the test population size was fixed at 99.

To assess the effect of marker density on the quality of prediction, evenly-distributed SNP subsets of different sizes were selected following the recommendation of Spindel et al. (2015). The number of markers was varied in each repetition as follows: 9824, 2945, 1473, 982, 737, 589, 491, 421, and 369; 9824 corresponds to the total number of SNP markers and 2945 corresponds to the number of markers representative of single bins on the consensus map (Tayeh et al., in press). The marker with the highest minor allele frequency (MAF) within each bin was selected. Smaller marker subsets, viz. 1473–369, were obtained out of the 2945-marker list: a single marker was retained every 2, 3, 4, 5, 6, 7, or 8 successive bins, respectively.

Predictions based on randomly-sampled training sets were also compared with those obtained from optimized training sets using the method proposed by Rincent et al. (2012). Comparisons were made for all traits with different training set sizes (240, 210, 180, 150, 120, 90, 60, 30, and 15 accessions) following 50 repetitions. As described above, the same test set (*n* = 99 accessions) was used for all training set sizes within each repetition. Each optimized training set was generated after 2000 iterations devoted to maximize the expected reliability of predictions from an initial randomly-sampled set. Accessions were replaced iteratively in the training set ongoing improvement. For a training set size of 240, and as in Rincent et al. (2012), the mean of the generalized coefficients of determination of the contrast (CDs) between each accession not included in the training set and the mean of the complete CRB339 collection, referred to as CDmean, was calculated at each iteration. The training set corresponding to the highest CDmean across all iterations was finally retained. For smaller training sets (210–15 accessions), CDs were calculated between each accession not included neither in the training set nor in the test set and the mean of the CRB339 collection. Variance ratios (λ; Rincent et al., 2012) of 0.01 or 0.4 were tested to calculate CDs as heritabilities for TSW, NSeed, and BegFlo from the 2007 field trial were 0.98, 0.71, and 0.99, respectively (Burstin et al., 2015).

In order to evaluate the performance of the genomic prediction, the following accuracy statistics were calculated for each trait: (i) the phenotypic prediction accuracy, expressed as the Pearson correlation between phenotypic data obtained for the test set in years 2003 and 2007, and (ii) the genomic prediction accuracy, expressed as the Pearson correlation between GEBVs estimated for the test set and the observed phenotypic values. Besides accuracies, three other parameters were calculated: the *R*^2^ and *Q*^2^ coefficients, evaluating the goodness of fit of the model to data from the training and test sets, respectively, and the MSEP (Burstin et al., 2015). The mean over all repetitions and standard deviation were calculated for each parameter in each experiment.

As predictions using training and test sets' data from the same year or the same population may bias the evaluation of the model performance, and in order to meet realistic conditions, genomic predictions were undertaken across years (2003 and 2007 or 2007 and 2011) and/or across populations. In this latter case, the CRB339 collection was taken as a training set and the 746-RIL population (Supplementary Table 2) as a test set. Only SNP markers polymorphic in at least one RIL bi-parental population were used in the cross-population experiment (*n* = 9700 out of 9824 markers; Supplementary Table 3). Prediction models were trained with the five above-cited statistical methods using, as training populations, all or subsets (250, 150, or 50 accessions) of the genetic resource collection. The sampling of the training sets comprising 250, 150, or 50 accessions was made using the CDmean-based method (λ = 0.01) with modifications: only the contrast between each accession not included in the training set and the complete CRB339 collection was considered for the selection of the optimized training set; the contrast with RIL populations was not taken into account by the applied script.

## Results

**Figure 1:**
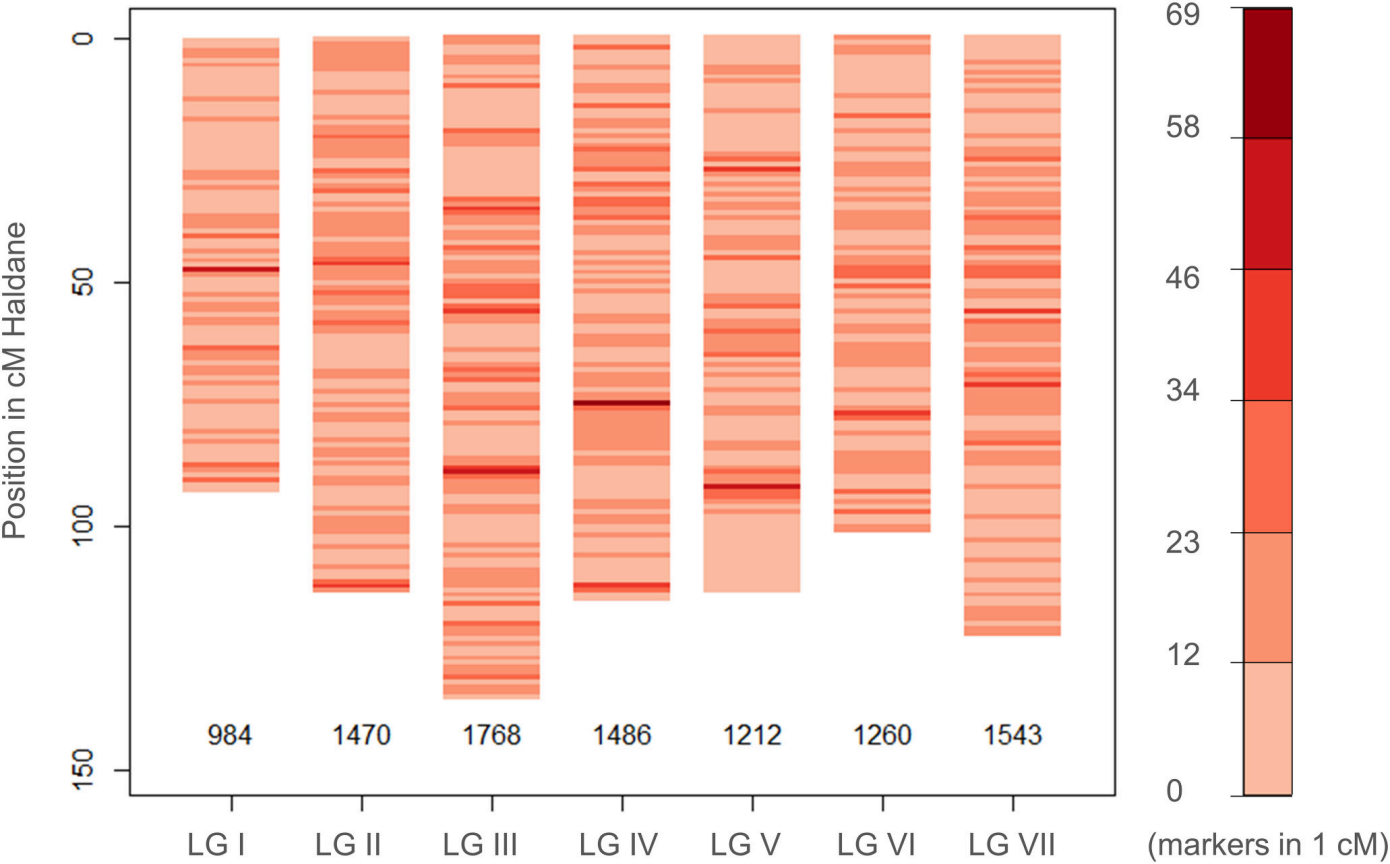
**Distribution of the SNP markers used to genotype the CRB339 collection on the pea consensus genetic map**. Information on the genetic positions of 9723 SNP markers are available and presented (see Tayeh et al., in press; and Supplementary Table 3).

**Figure 2:**
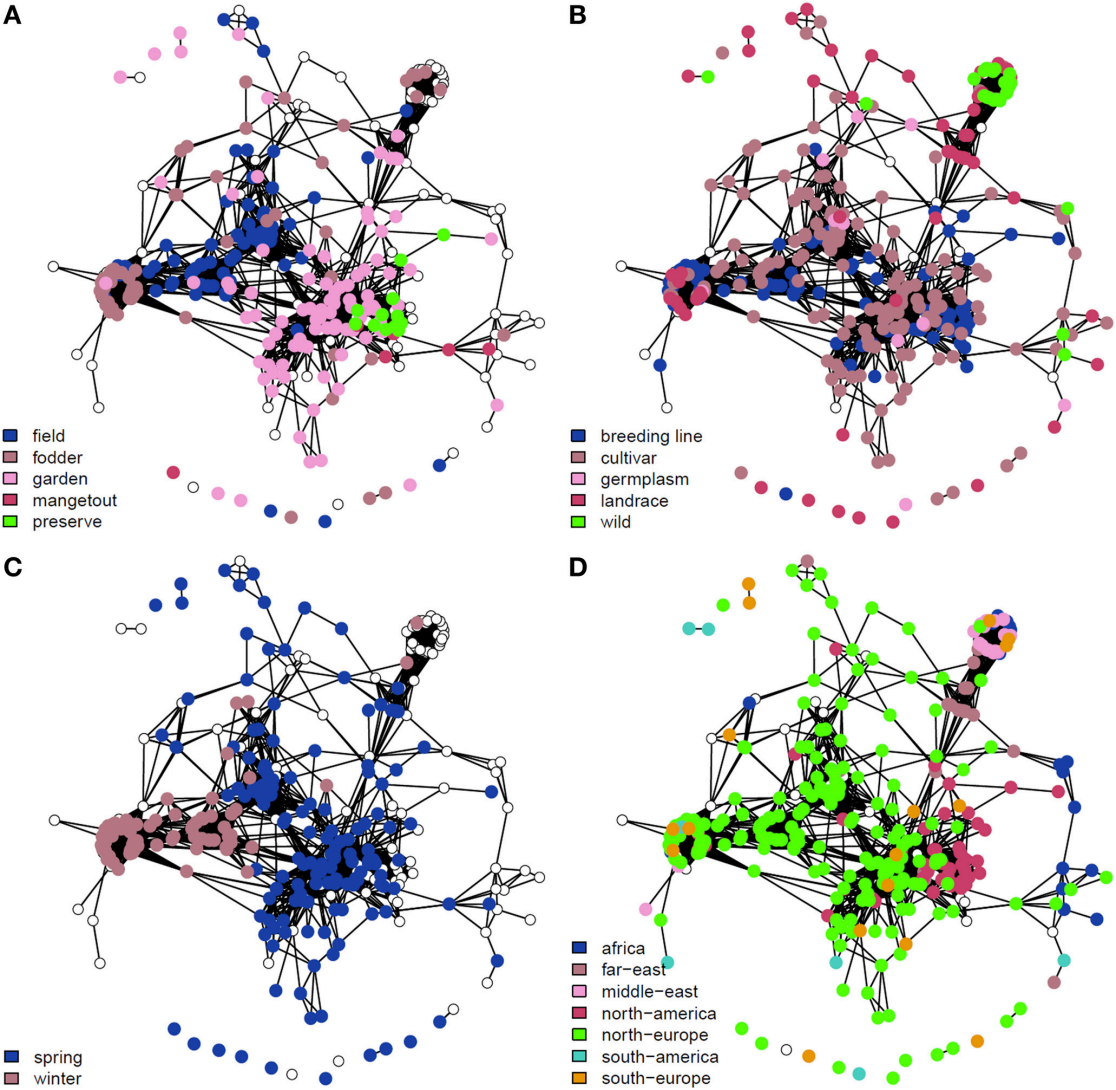
**Genetic dissimilarity amongst the 339 pea accessions composing the genetic resource collection**. Two-dimensional networks were constructed using the R package, network (Butts, 2008). Each accession is represented by a dot. Pairs of accessions with a genomic relationship coefficient >0.2 are linked. Major classes for the use type **(A)**, the population type **(B)**, the sowing type **(C)**, and the geographic origin **(D)** are color-coded. Uncolored dots correspond to accessions that were not assigned to any of the classes.

The newly-developed GenoPea 13.2k SNP array (Tayeh et al., in press) was successfully used to genotype a collection of 339 diverse pea accessions, the CRB339 collection (Supplementary Table 1). Of the 9997 SNP markers reported to be of high-quality across all 12 bi-parental RIL populations in Tayeh et al. (in press), 9824 markers also exhibited high-quality clustering (score 1; Supplementary Figure 1) when used to genotype this collection (see Materials and Methods; Supplementary Tables 1, 3). The percentage of missing data was 0.28% and 483 (4.83%) markers had a MAF below 5% (Supplementary Table 3). Of the total 9824 markers, 9723 were placed on the consensus map from Tayeh et al. (in press; Supplementary Table 3). As shown in Figure 1, these markers cover the seven pea LGs with 984 (LG I) up to 1768 (LG III) markers per LG and a mean density per cM ranging between 10.56 (LG I) and 13.06 (LG III) markers. Figure 2 illustrates the genetic relationships among the accessions composing the CRB339 collection as revealed by the GenoPea 13.2k SNP array. A clustering according to passport data (Figure 2 and Supplementary Table 1) was generally noted and few accessions were isolated. A principal coordinates analysis was also implemented to evaluate the genetic diversity in the collection under scrutiny: 26.3% of the genetic variance was explained by the two first principal components (Supplementary Figure 2). For all LGs, LD in the CRB339 collection rapidly declined with increasing genetic distances (Supplementary Figure 3); LD decayed below *r*^2^ = 0.2 at less than (or around) 0.5 cM.

A wide range of phenotypic variation was observed for TSW, NSeed, and BegFlo: in 2007, TSW, NSeed, and BegFlo ranged between 35.6 and 472.5 g, 11.4 and 268.6 seeds, and 540 and 1301 degrees × days, respectively. Broad-sense heritabilities were estimated at 0.98 (TSW), 0.71 (NSeed), and 0.99 (BegFlo; Burstin et al., 2015). The highest within-year within-population mean genomic prediction accuracies, according to the applied statistical methods (see Materials and Methods) and following 200 cross-validations with 240-accession randomly-selected training sets, reached 0.86 for TSW, 0.73 for NSeed, and 0.79 for BegFlo (Table 1 and Supplementary Table 4). Except for TSW, genomic prediction accuracies were equal or higher than phenotypic prediction accuracies calculated as the mean of the Pearson correlation between phenotypic values from 2003 to 2007 (See Materials and Methods; Table 1). The predictive performances of the kPLSR, GBLUP, BayesA, and BayesB methods were almost equivalent whereas LASSO performed less well (Table 1). No significant difference in accuracy could be noted whether markers with low MAF were considered to train the prediction model or not (Supplementary Table 4).

**Table 1:** **Prediction accuracy of TSW, NSeed, and BegFlo within the CRB339 collection**.

		**Phenotypic prediction accuracy**	**Genomic prediction accuracy 2007_2007**					**Genomic prediction accuracy 2007_2003**				
			**kPLSR**	**LASSO**	**GBLUP**	**BayesA**	**BayesB**	**kPLSR**	**LASSO**	**GBLUP**	**BayesA**	**BayesB**
TSW	Mean	0.93	0.86	0.82	0.86	0.86	0.86	0.83	0.79	0.82	0.83	0.83
	stdev	0.02	0.02	0.03	0.03	0.03	0.03	0.03	0.04	0.03	0.03	0.03
NSeed	Mean	0.69	0.73	0.66	0.73	0.73	0.72	0.67	0.64	0.67	0.68	0.67
	stdev	0.06	0.04	0.05	0.04	0.04	0.04	0.05	0.06	0.06	0.05	0.05
BegFlo	Mean	0.79	0.78	0.76	0.78	0.78	0.79	0.64	0.63	0.64	0.64	0.65
	stdev	0.05	0.04	0.04	0.04	0.04	0.04	0.05	0.06	0.05	0.05	0.05

Mean and standard deviation (stdev), across 200 repetitions, of the phenotypic prediction accuracy and of within- and across-year genomic prediction accuracies are provided for each trait.

**Figure 3:**
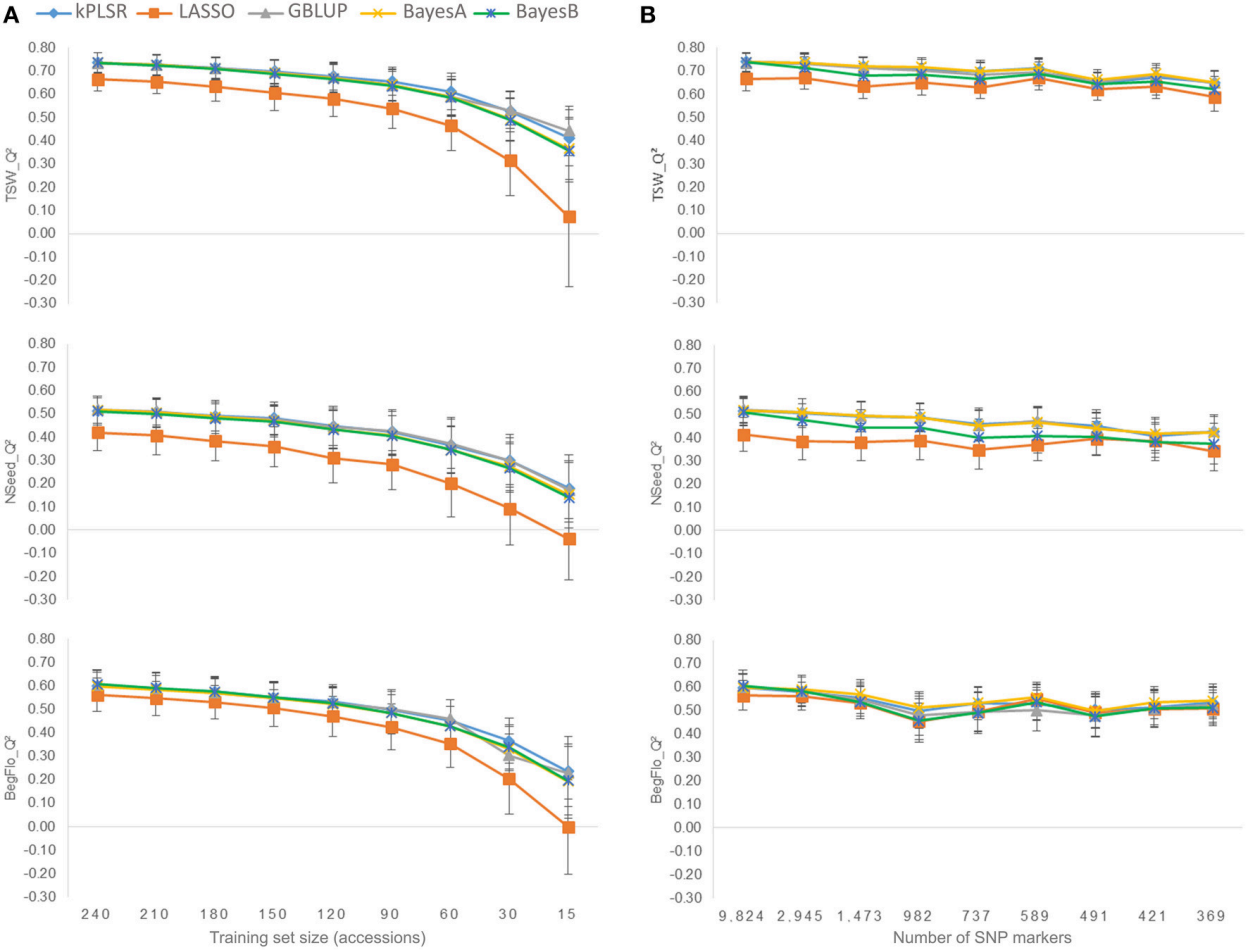
**Effect of the calibration set size (A) and marker density (B) on the prediction quality of TSW, Nseed, and BegFlo of the CRB339 collection**. Nine different tests per parameter were conducted. For each trait and each parameter, means and standard deviations of *Q*^2^ resulting from five statistical methods (see color codes) were used to construct the corresponding plot.

The quality of the prediction models (*Q*^2^) decreased with the reduction of the size of the training sets from 240 to 15 accessions (Figure 3A and Supplementary Table 4). Maximum *Q*^2^ with 240 and 15-accession training populations were 0.74 and 0.44 for TSW, 0.52 and 0.18 for NSeed, and 0.61 and 0.23 for BegFlo, respectively. kPLSR and GBLUP were the best methods especially with smaller training set sizes.

Decreasing the number of markers from 9824 to 2945 by retaining only a single marker per unique map position (see Materials and Methods) did not degrade the genomic prediction accuracies for any of the three traits (Figure 3B and Supplementary Table 5). *Q*^2^ slightly decreased when the number of markers was further reduced but less dramatically than in the case of training set size variation (Figure 3). Considering the best statistical method in each case, *Q*^2^ decay was 11.8% for TSW (BayesA), 17.3% for NSeed (kPLSR), and 9.3% for BegFlo (BayesA) when the marker number decreased from 9824 to 369. The best prediction models at low marker density were kPLSR, GBLUP, and BayesA.

**Figure 4:**
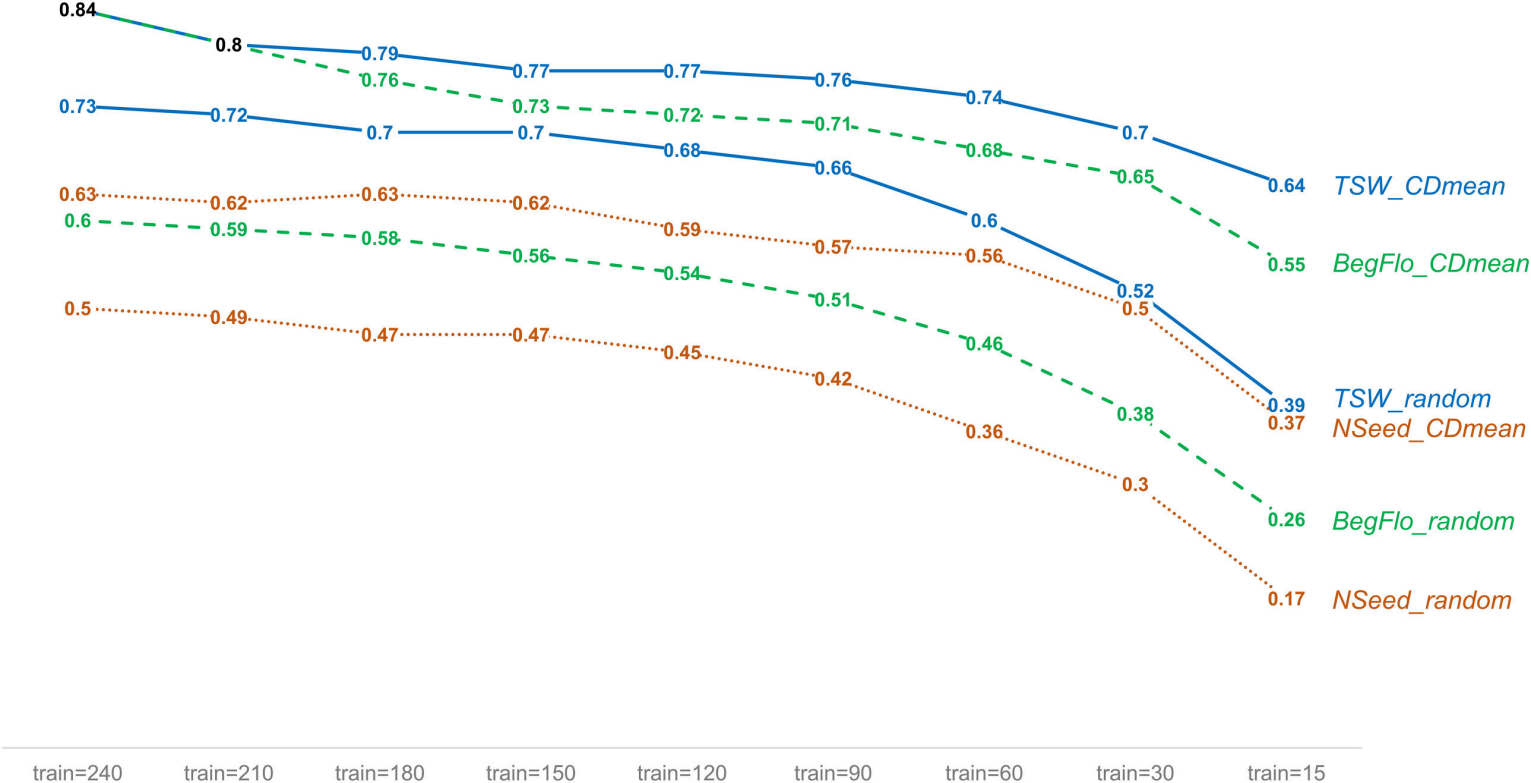
**Improvement of the prediction quality of TSW, NSeed, and BegFlo within the CRB339 collection with training sets sampled using the CDmean-based method**. For each trait, mean *Q*^2^ obtained with the kPLSR method for nine different training set (train) sizes are shown. Two methods to select the training sets were applied: random sampling and CDmean-based sampling (Rincent et al., 2012). Fifty repetitions were made in each case. Data from this figure were obtained using a variance ratio of 0.01 to calculate CD in the CDmean-based method.

The effect of the composition of the training set on the prediction quality was tested using training sets sampled either randomly or using an optimization protocol based on CDmean (Rincent et al., 2012). Different training set sizes were also considered (see Materials and Methods). The choice of the training set based on CDmean permitted a significant improvement in prediction accuracy and so, *Q*^2^ (Supplementary Table 6 and Supplementary Figure 4). For instance, a mean *Q*^2^ of 0.84 was obtained for BegFlo using optimized training sets of 240 accessions whereas mean *Q*^2^ did not exceed 0.6 with randomly-sampled training sets of the same size (Figure 4 and Supplementary Table 6). Higher prediction quality was notable whatever the training set size was and particularly in the case of training sets of reduced sizes (Figure 4, Supplementary Figure 4 and Supplementary Table 6). No difference could be observed when using a variance ratio λ of either 0.01 or 0.4 to calculate CD according to the method proposed by Rincent et al. (2012; Supplementary Table 6 and Supplementary Table 7).

**Figure 5:**
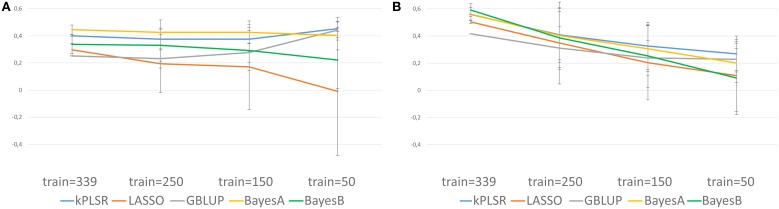
**Illustration of the quality of genomic predictions of TSW (A) and BegFlo (B) of RILs from nine populations based on models trained on 339, 250, 150, or 50 accessions from the CRB339 collection**. Five statistical methods were applied and genotypic information from 9700 SNP markers were considered. Mean Q^2^ and standard deviations used to construct this figure were obtained based on cross-validations with 50 repetitions.

Cross-predictions using models trained on the CRB339 collection to predict the performances of 746 RILs arising from nine crosses, all having both parental lines included in the genetic resource collection (Supplementary Table 1 and Supplementary Table 2) were also undertaken. Genotypic information were obtained from 9700 common high-quality SNP markers (Supplementary Table 3). Furthermore, phenotypic data for the CRB339 collection and the RILs were scored at the same location but in different years: 2007 and 2011, respectively. NSeed could not be successfully cross-predicted (Supplementary Table 8). For TSW and BegFlo, when the complete CRB339 collection was included in the training set, maximum mean *Q*^2^ of 0.44 for TSW with BayesA and 0.59 for BegFlo with BayesB were obtained (Figure 5 and Supplementary Table 8). In case of TSW, *Q*^2^ greatly to slightly increased or decreased with the reduction of the training set size from 339 to 50 accessions, depending on the statistical algorithm (Figure 5A). For BegFlo, *Q*^2^ decreased with all algorithms; *Q*^2^ decay was 45.2% with GBLUP and reached 84.7% with BayesB (Figure 5B). kPLSR and BayesA were consistently the best methods for TSW and BegFlo prediction (Figure 5 and Supplementary Table 8).

## Discussion

GS is currently revolutionizing plant breeding with a promise for significant gain per unit time and cost in crop improvement programs (Heffner et al., 2010, 2011) and is experiencing an intense scientific research activity. Genomic prediction studies in plant species including wheat (Heffner et al., 2011; Poland et al., 2012; Heslot et al., 2013; Charmet et al., 2014), barley (Lorenz et al., 2012), maize (Rincent et al., 2012; Massman et al., 2013), rye (Wang et al., 2014), rice (Iwata et al., 2015; Spindel et al., 2015), sugarcane (Gouy et al., 2013), loblolly pine (Resende et al., 2012b), eucalyptus (Resende et al., 2012a), pea (Burstin et al., 2015), and soybean (Jarquín et al., 2014) have been published in the past 5 years. But, for most of these species, especially pea, efforts are still needed to provide a full insight into the prospects of GS for polygenic traits of agronomic interest and the factors that determine its success. In a previous paper, the genomic predictability of pea TSW, NSeed, and BegFlo was inspected with a limited number of SNP markers and have shown encouraging accuracies (Burstin et al., 2015). One of the objectives of the present work was to assess the genomic prediction accuracy of the same traits when effects of up to 9824 SNP markers can be estimated. As in Burstin et al. (2015), TSW was better predicted than BegFlo and NSeed. When the GEBVs from 2007 were correlated to phenotypes from 2003, to avoid any inflation effect related to genotype-by-year interactions (Burstin et al., 2015), genomic prediction accuracies were high for all traits and reached 0.83 for TSW, 0.68 for NSeed, and 0.65 for BegFlo (Table 1). Within- and cross-environment prediction accuracies (Table 1 and Supplementary Table 4) were sufficiently high to merit considering the implementation of GS in pea breeding programs, especially given the availability of a high-density SNP genotyping platform such as the GenoPea 13.2K SNP Array (Tayeh et al., in press), decreasing genotyping costs, and static or increasing phenotyping costs (Jannink et al., 2010). According to the literature, the genomic prediction depends on many factors that affect more or less importantly the final accuracy. Besides the heritability and the architecture of the trait, the training population size, the relatedness between individuals from the training and test populations, the LD decay rate, the genotyping platform, the genetic marker density, and the statistical prediction method are all influencing factors. The impact of some of these on the genomic prediction in pea was evaluated in this study and results are discussed below.

### The statistical prediction method

Independently of the training population size or the marker number, and except for LASSO, the prediction accuracy within the CRB339 collection was not or only modestly influenced by the choice of the statistical method (Figure 3). A similar conclusion was reached in previous studies in different plant species (Heffner et al., 2011; Lorenz et al., 2012; Resende et al., 2012b; Gouy et al., 2013; Charmet et al., 2014; Jarquín et al., 2014) while using multiple models for comparison. LASSO performed differently than other methods: it has generally shown an inferior performance for all traits and conditions (Figure 3). The inferior performance of LASSO was also underlined in Burstin et al. (2015). This suggests that either the utilization of the LASSO requires optimization to ensure a better selection of its variables (and so of the most relevant SNPs) or that the method is not adequate for the studied traits possibly being not controlled by major genes. kPLSR was not widely used in genomic prediction so far; it was only applied to predict the grain shape in rice (Iwata et al., 2015) according to the authors of the corresponding study. As in Iwata et al. (2015), this method yielded the largest *Q*^2^ values in most cases and appeared as a method of choice for future applications in pea. Contrary to within-population predictions, the effect of the statistical methods on the prediction accuracy was more pronounced in cross-population predictions, especially in case of TSW (Figure 5). Again, kPLSR, with BayesA, yielded the highest accuracies across all training set sizes.

### The genotyping platform and marker density

The prediction quality, expressed as *Q*^2^, attained 0.74 for TSW, 0.52 for NSeed, and 0.60 for BegFlo with BayesA and thus exceeded values obtained with the genetic resource collection in Burstin et al. (2015) where less marker density was used: the number of markers was increased from 331 SNP markers in Burstin et al. (2015) to 9824 SNPs in this study. *Q*^2^ values for TSW, NSeed, and BegFlo were 21.3, 92.6, and 33.3% higher in this study, respectively, using BayesA (Supplementary Table 5) and were still 6.6, 55.6, and 20% higher when the number of markers was reduced to 369 (Supplementary Table 5) which is equivalent to the number from Burstin et al. (2015). This suggests that the gain in accuracy observed for all three traits using either all (*n* = 9824) or just a subset (*n* = 369) of the SNP markers from the GenoPea 13.2K SNP Array was mainly due to the even distribution of markers across LGs (Figure 1). Similarly, higher accuracies were obtained with markers derived from the genotyping-by-sequencing platform compared to markers from the Diversity Array Technology platform in two sets of wheat breeding lines (Poland et al., 2012; Heslot et al., 2013). These were not simply due to higher marker density. Even with a comparable number of markers, the genotyping-by-sequencing platform led to a significant gain in accuracy for three out of the eight total traits predicted in Poland et al. (2012) and Heslot et al. (2013). Results from the present study demonstrated that: (i) the GenoPea 13.2K SNP Array is a valuable source of markers for GS programs; (ii) using the full number of markers (9824) do not bring any noise related to marker position/redundancy as conserving only one marker per bin position yielded similar or slightly lesser accuracies (Figure 3B); and (iii) despite the high LD decay rate over the pea LGs (Supplementary Figure 3), a limited number of evenly-distributed markers permit to predict important traits such as TSW, NSeed, and BegFlo with remarkable accuracies, maybe due to the structure of the collection. A limited impact of reducing the number of markers on prediction accuracy was also observed in wheat; only 10% accuracy loss was noted when reducing the number of markers from 1158 to 192 (Heffner et al., 2011). Likewise, on average, reducing marker number, respectively, from 73147 to 7142 and from 1023 to 384 did not reduce accuracy in a six-row barley breeding germplasm (Lorenz et al., 2012) and in a collection of advanced inbred rice breeding lines (Spindel et al., 2015).

### The training population size and composition

Reducing the training set size up to 15 accessions resulted in a dramatic decrease in the prediction accuracy of all three traits in this study (Figure 3A and Supplementary Table 4). *Q*^2^ decreased of 0.06–0.08 for TSW (7.9–12.6%), 0.07–0.11 for NSeed (12.9–26%), and 0.07–0.09 for BegFlo (11.2–16.3%) when the population size was reduced two-fold from 240 to 120 accessions. *Q*^2^ decays were at least 39.7 (TSW, GBLUP), 65.2 (NSeed, kPLSR), and 61% (BegFlo, kPLSR) when comparing results from experiments using 240 and 15-accession training sets. Similar observations were previously made in dairy cattle (VanRaden et al., 2009) or other plant (Heffner et al., 2011) GS experiments indicating that the reduction of the training population size negatively affect the estimation of marker effects and significantly deteriorate the prediction quality. Optimizing the sampling of the training population through the CDmean-based method proposed by Rincent et al. (2012) significantly improved the prediction accuracy for all traits and with all training set sizes (Figure 4 and Supplementary Figure 4). This method guarantees the selection of the less-related individuals in the training set while considering the whole network of kinship thus maximizing the relatedness between individuals in the training and test sets (Rincent et al., 2012). Taken altogether, these results indicated that the training set sampling based on CDmean is an important step to include in future GS schemes and also pointed out the fact that large training populations would be necessary to guarantee maximum accuracies. With SNP-rich genotyping tools becoming available, such as the GenoPea 13.2K SNP Array, creating a public database where all users of these resources could deposit genotyping data may be very useful for future genomic prediction efforts by the pea community. Limitation, however, would come from phenotyping. International projects devoted to large-scale phenotyping experiments should enhance publically accessible data. With such tools available, custom training sets would be easily selected by each partner to retrain the model appropriate for his population candidate to selection. Such efforts should be coupled with initiatives to develop statistical methods that take into account the genotype-by-environment interactions.

### Accuracy in cross-population prediction

When applied, GS will be used to predict the phenotypes of new breeding lines rather than perform cross-validations on individuals from the same panels from which the training sets were sampled. It was therefore of interest to assess the potential of the models trained on the CRB339 collection to predict the phenotypes of advanced RILs especially as parents of all nine RIL populations were included in the genetic resource collection. Similar cross-validation experiments are described in the literature with contradictory results. While cross-validation between sugarcane panels from Reunion and Guadeloupe yielded promising correlations ranging from 0.13 to 0.55 (depending on the trait; Gouy et al., 2013), models trained on one wheat population did not predict phenotypes in a different population or if so, low accuracies were noted (Charmet et al., 2014). Close-to-zero prediction accuracies were obtained when a model developed for eucalyptus CENIBRA population was used to predict growth and wood quality trait phenotypes in FIBRIA population or vice versa (Resende et al., 2012a). Average *Q*^2^ values obtained by cross-population cross-validation for TSW and BegFlo in this study were relatively high. Considering the training set of maximum size (*n* = 339 accessions), maximum mean *Q*^2^ was 0.44 for TSW (BayesA) and 0.59 for BegFlo (BayesB). However, it was -0.16 for Nseed (LASSO). This is consistent with high heritability and less genotype-by-environment interaction reported for TSW and BegFlo than for NSeed (Burstin et al., 2015). Moreover, exceptional environmental conditions affecting yield-related traits may have occurred in 2011. Correlations of estimated GEBVs with phenotypic data from other environments should be next examined in an attempt to shed light on this issue.

In conclusion, prediction results were very promising for future implementations of GS in pea breeding programs provided that the training populations will be chosen carefully and that the relatedness between training and test sets will be optimized. The GenoPea 13.2K SNP Array (Tayeh et al., in press) appeared to provide enough high-quality markers for genomic prediction. Genomic predictions were evaluated on an individual trait basis in this study, while a breeder's goal is to improve an overall performance (net merit), and so a combination of economically- and agriculturally-important traits. It will thus be interesting to define economic indices combining the most important pea traits and assay cross-population cross-prediction based on the net merit. More traits, populations, and environments are also to be considered in the future.

### Conflict of interest statement

The authors declare that the research was conducted in the absence of any commercial or financial relationships that could be construed as a potential conflict of interest.

## Supplementary material

Supplementary Table 1 **List of the 339 pea accessions composing the genetic resource collection used in this study**. See Burstin et al. (2015) for additional information. Data regarding the parents of RIL populations are shaded in blue.

Supplementary Table 2 **Description of the 9 bi-parental RIL populations representing the test set used in the cross-population cross-validation experiment**.

Supplementary Table 3 **Full list of SNP markers used in genomic prediction**. The complete set of markers for the CRB339 collection comprised all 9,824 SNP markers. Markers used in the cross-population cross-prediction experiment between the genetic resource collection and RIL populations are in bold. The polymorphic RIL population(s) for each marker is(are) indicated. Full marker description is available in Tayeh et al. (in press).

Supplementary Table 4 **Impact of the size of the training population on the performance of kPLSR, LASSO, GBLUP, BayesA, and BayesB to predict TSW, NSeed, and BegFlo in the CRB339 collection**. The predictive performances of the statistical methods were assessed under two conditions: A, including all 9,824 SNP markers, and B, excluding markers with MAF below 5%. Two hundred repetitions were applied to assess the quality of the trained models in each case and mean values for the different prediction evaluation parameters are indicated. Standard deviations (stdev) are provided.

Supplementary Table 5 **Impact of the genetic marker density on the performance of kPLSR, LASSO, GBLUP, BayesA, and BayesB to predict TSW, NSeed, and BegFlo in the CRB339 collection**. Two hundred repetitions were applied to assess the quality of the trained models in each case and mean values for the different prediction evaluation parameters are indicated. Standard deviations (stdev) are provided.

Supplementary Table 6 **Comparison of the predictive performances of kPLSR, LASSO, GBLUP, BayesA, and BayesB with training sets sampled either randomly or based on CDmean**. Nine different training set (train) sizes were considered. All three phenotypic traits, viz. TSW, NSeed, and BegFlo, were predicted following 50 repetitions within the CRB339 collection. A variance ratio of 0.01, corresponding to a heritability nearing 1, was used for the CDmean-based method. Mean and standard deviation values for the different prediction evaluation parameters are provided.

Supplementary Table 7 **Comparison of the predictive performances of kPLSR, LASSO, GBLUP, BayesA, and BayesB with training sets sampled either randomly or based on CDmean**. Nine different training set (train) sizes were considered. All three phenotypic traits, viz. TSW, NSeed, and BegFlo, were predicted following 50 repetitions within the CRB339 collection. A variance ratio of 0.4, corresponding to a heritability of ca. 0.71, was used for the CDmean-based method. Mean and standard deviation values for the different prediction evaluation parameters are provided.

Supplementary Table 8 **Quality of cross-population prediction of TSW, NSeed, and BegFlo**. The CRB339 collection and RIL populations were used as training and test sets, respectively. Mean *Q*^2^ and standard deviation (stdev), across 50 repetitions, are provided for each trait, statistical prediction method, and training set size.

Supplementary Figure 1 **Examples of GenoPlots observed after genotyping a large pea genetic resource collection, including the CRB339 collection, using the GenoPea 13.2K SNP Array**. Each genotype sample is represented by a dot. Genotypes are called using their signal intensity (Norm R) and allele frequency (Norm Theta) relative to canonical cluster positions. Color codes in the graphics refer to: red, AA (homozygous); purple, AB (heterozygous); and blue, BB (homozygous) genotypes. The number at the top right of each GenoPlot indicates the score given for all SNPs showing similar number and position of clusters.

Supplementary Figure 2 **Principal coordinates analysis on the distance matrix of the CRB339 collection illustrating dissimilarity amongst accessions**. Major classes for the use type **(A)**, the population type **(B)**, the sowing type **(C)**, and the geographic origin **(D)** are color-coded.

Supplementary Figure 3 **Linkage disequilibrium in the CRB339 collection**. Each plot represents a single LG where pairwise *r*^2^ values (Y-axis) and genetic distances in cM Haldane between all markers (X-axis) were plotted. Regression lines in red are based on Hill and Weir (1988). The plots displayed in small windows are zoom views of LD decay for maximum inter-marker distance of 10 cM. Red vertical lines were drawn at 0.5 cM.

Supplementary Figure 4 **Improvement of the prediction quality of TSW (A), Nseed (B), and BegFlo (C) in the CRB339 collection with training sets sampled using the CDmean-based method**. Results shown were obtained with the kPLSR method. Different training set sizes (train) were used: 240, 210, 180, 150, 120, 90, 60, 30, and 15 accessions. Boxplots illustrate the distribution of the *Q*^2^ values from 50 repetitions; mean values are indicated in red above each boxplot.
